# Genome Assembly and Transcriptome Analysis of the Fungus *Coniella diplodiella* During Infection on Grapevine (*Vitis vinifera* L.)

**DOI:** 10.3389/fmicb.2020.599150

**Published:** 2021-01-11

**Authors:** Ruitao Liu, Yiming Wang, Peng Li, Lei Sun, Jianfu Jiang, Xiucai Fan, Chonghuai Liu, Ying Zhang

**Affiliations:** ^1^Zhengzhou Fruit Research Institute, Chinese Academy of Agricultural Sciences, Zhengzhou, China; ^2^The Key Laboratory of Plant Immunity, College of Plant Protection, Nanjing Agricultural University, Nanjing, China

**Keywords:** *Coniella diplodiella*, grapevine, genome assembly, transcriptome, pathogenicity, effector

## Abstract

Grape white rot caused by *Coniella diplodiella* (Speg.) affects the production and quality of grapevine in China and other grapevine-growing countries. Despite the importance of *C. diplodiella* as a serious disease-causing agent in grape, the genome information and molecular mechanisms underlying its pathogenicity are poorly understood. To bridge this gap, 40.93 Mbp of *C. diplodiella* strain WR01 was *de novo* assembled. A total of 9,403 putative protein-coding genes were predicted. Among these, 608 and 248 genes are potentially secreted proteins and candidate effector proteins (CEPs), respectively. Additionally, the transcriptome of *C. diplodiella* was analyzed after feeding with crude grapevine leaf homogenates, which reveals the transcriptional expression of 9,115 genes. Gene ontology enrichment analysis indicated that the highly enriched genes are related with carbohydrate metabolism and secondary metabolite synthesis. Forty-three putative effectors were cloned from *C. diplodiella*, and applied for further functional analysis. Among them, one protein exhibited strong effect in the suppression of BCL2-associated X (BAX)-induced hypersensitive response after transiently expressed in *Nicotiana benthamiana* leaves. This work facilitates valuable genetic basis for understanding the molecular mechanism underlying *C. diplodiella*-grapevine interaction.

## Introduction

Grapevine (*Vitis vinifera* L.) is an economically important fruit crop around the world. However, the widely planted *Vitis vinifera* cultivars are highly susceptible to various fungal pathogens (Armijo et al., 2016). Among them, the pathogenic fungus *Coniella diplodiella* (Speg.) Petr. and Syd, which causes white rot disease on the grapevine, can severely affect vine growth and grape production with an annual yield loss of 10–20% (Li et al., 2008). *Coniella diplodiella* is a necrotrophic fungal pathogen, which infects the injured grape berries, spikelets, young branches and leaves under high temperature and high humidity environments (Chethana et al., 2017; Zhang et al., 2017b). Several antifungal chemicals were used to control the white rot disease, however, the continuous application of chemical fungicides lead to the emergence of resistant pathogens and finally food safety and environmental problems (Han et al., 2015; Escribano-Viana et al., 2018).

Although the application of the fungicides is still one of the most effective ways to combat the *C. diplodiella*, efforts have been applied on the genetic basis of pathogen resistance and biological control reagents in recent years. Several candidate pathogenesis-related (PR) genes have been identified by comparative transcriptome analysis of susceptible and resistant grapevine species or cultivars challenged by *C. diplodiella* (Su et al., 2019; Zhang et al., 2019). Both salicylic acid (SA) and jasmonic acid (JA) synthesis signaling pathways may involve in host resistance against *C. diplodiella*. Association mapping in Chinese wild grapevines was performed to investigate potential quantitative trait loci (QTLs) involved in white rot disease resistance (Zhang et al., 2017c). However, molecular mechanism underlying pathogenicity of *C. diplodiella* on grapes has not been well understood so far, possibly due to the lack of genome information of this species.

The genus *Coniella* contains plenty of plant pathogens of economically important agricultural crops, including *Vitis*, *Fragaria*, and *Punica* (Alvarez et al., 2016). These *Coniella* species cause foliar, fruit, stem, and root diseases, bringing huge economic loss. However, the understanding of their pathogenicity mechanisms is very limited as there are no relevant genetic data available for these pathogens. Recently, the draft genome of *Coniella lustricola*, a new *Coniella* species isolated from submerged detritus, was reported (Raudabaugh et al., 2017). With the advantage of next generation sequencing (NGS) technologies, the number of sequenced fungal pathogen genomes are increasing rapidly. Genome annotation and comparison reveals the infection mechanism of plant pathogens of economically important crops, providing the genetic basis for understanding the plant-pathogen arm races (Moller and Stukenbrock, 2017; Raudabaugh et al., 2017).

Pathogens use secreted effectors to interfere with plant immunity, which are determinants of host-pathogen interaction (Dou and Zhou, 2012). Although some effectors have been characterized from the biotrophic and hemibiotrophic fungal pathogens, knowledge on the roles of effectors encoded by the necrotrophic fungi is still very limited (Lo Presti et al., 2015; Franceschetti et al., 2017). In addition to function as key virulence factors, effectors can be used for probing plant germplasm to seek resistance (R) genes in disease-resistant species or disease susceptibility (S) genes in disease-prone species (Vleeshouwers and Oliver, 2014; Xu et al., 2019). Wild Chinese *Vitis* species contain abundant and diverse gene resources for the genetic improvement of grapevine (Wan et al., 2007; Li et al., 2008). We have identified several grapevine accessions with conferred resistance to white rot disease (Zhang et al., 2017b). Furthermore, transcriptome analysis of the leaves of the resistant wild grapevine species *Vitis davidii* and the susceptible cultivar of *V. vinifera* “Manicure Finger” challenged by *C. diplodiella* identified more than 20 disease resistance-related genes (Zhang et al., 2019). However, the molecular mechanisms underlying the interaction of *C. diplodiella* with its host are still scarce.

In order to attain genetic information on the grape white rot fungus *C. diplodiella*, we sequenced the genome of *C. diplodiella* in this study. The 40.93 Mb genome contains 9,403 predicted genes coding for a large number of pathogenicity-related genes, including carbohydrate-active enzymes, secondary metabolite synthesis, effectors, and so on. These genes were differentially regulated by susceptible and resistant grapevine varieties. A preliminary screening of putative effector proteins revealed that one effector involved in the suppression of BCL2-associated X (BAX)-induced HR in tobacco, which may also important for the interaction between grapevine and *C. diplodiella*. Taken together, our results will improve the understanding of the pathogenicity of *C. diplodiella* on grapevine, and provide genome information for further comparison with other plant pathogens of the *Coniella* genus.

## Materials and Methods

### Fungal Strain and DNA Extraction

The *Coniella diplodiella* (Speg.) strain WR01 (from the Institute of Plant Protection, Chinese Academy of Agricultural Sciences) was cultured on PDA (Potato Dextrose Agar) medium at 28°C. Total DNA of *C. diplodiella* was isolated from the mycelia using QIAGEN^®^ Genomic DNA kit following the standard procedures.

### Genome Sequencing and Assembly

Qualified DNA was sheared using Covaris g-TUBE device. The fragmented DNA was repaired using PacBio Template Prep Kit (Pacific Biosciences, United States). The enrichment for long fragments was done by BluePippin size selection system (Sage Science) to construct a 20 kb library. After DNA purification using AMPure^®^ PB beads (Pacific Biosciences, United States), the DNA fragments were ligated to the hairpins (SMRTbell^TM^ templates). The library quality was checked by Agilent Bioanalyzer 2100 (Agilent Technologies, CA, United States) and Qubit 2.0 Fluorometer (Invitrogen, Life Technologies, CA, United States). The prepared SMRTbell^TM^ templates was bound with magbead and loaded on a SMRT cell of PacBio Sequel platform. Single-molecule real-time (SMRT^®^) DNA sequencing (Berlin et al., 2015) was performed in Nextomics Biosciences Co., Ltd (Wuhan, China) according to the manufacturer’s protocol (Pacific Biosciences, CA, United States). Raw reads were processed by the SMRT Link v2.3.0 in the default mode to remove the adaptor sequences and low quality reads (below quality 0.8), and the filtered reads (6.3 G) were assembled to contigs with no gaps by CANU with default parameters (Koren et al., 2017). The sequencing data generated were deposited in NCBI Short Read Archive database (SRA accession: PRJNA649095).

### Repetitive Elements and Non-coding RNA Analysis

Repetitive sequences were identified by aligning the assembly sequences with the known sequences in the Repbase database using RepeatProteinMasker and RepeatMasker (Bao et al., 2015) softwares, or by *de novo* prediction using three tools including RepeatModeler, LTRfinder (Xu and Wang, 2007), and PILER (Edgar and Myers, 2005) with default parameters. Tandem Repeats Finder (Benson, 1999) and MIcoSAtellite (Beier et al., 2017) were used to identify tandem repeat sequences and simple sequence repeats (SSR), respectively. tRNA sequences and its secondary structures were predicted using tRNAscan-SE (Schattner et al., 2005) software. rRNAs were predicted by RNAmmer (Lagesen et al., 2007) software.

### Gene Prediction and Annotation

Protein coding gene models were predicted from the repeat-masked genome using three approaches: (1) *ab initio* gene prediction by SNAP (Korf, 2004) and Augustus (Stanke et al., 2004) using a transcriptome-based training set constructed by PASA (Haas et al., 2003); (2) protein homology-based prediction, where tBLASTn and GeneWise (Ter-Hovhannisyan et al., 2008) were used to align protein sequences from related species to the assembled genome; (3) transcriptome-based prediction, where transcripts were aligned to assembled genome using Exonerate software. Finally, EVidenceModeler (EVM) program was used to integrate all predicted gene models into a weighted consensus gene set (Haas et al., 2008).

All predicted gene models were annotated functionally by using several databases, including NCBI Non-Redundant (NR) and Swiss-Prot protein databases, Cluster of Orthologous Groups of proteins (COG), and Kyoto Encyclopedia of Genes and Genomes (KEGG) using BLASTp with *E*-value cut-off of 1e–05. Gene Ontology (GO) was performed using InterProScan.

### Comparative Genomics

Orthologous groups of *C. diplodiella* and 12 other fungal genomes were determined by OrthoMCL (Li et al., 2003) software executed with the All-vs.-all BlASTp search with an *E*-value threshold of 1e–05. Amino acids of single-copy ortholog genes were aligned using MAFFT (Katoh and Standley, 2013). Gaps present in the alignments were removed by Gblocks (Castresana, 2000). The phylogenetic tree was constructed using RaxML (Stamatakis, 2014) with the GTRGAMMA model and 100 bootstrap replications, based on the concatenated alignments of single-copy ortholog families. Synteny analysis of *C. diplodiella* and its relative *Coniella lustricola* was performed by MUMmer (Kurtz et al., 2004). Mummerplot was used to produce the dot-blot of the MUMmer alignments.

### Functional Annotation of Specific Gene Categories

Secretome was predicted using a combination of several tools. Signal peptides were predicted by both SignalP v4.1 (Petersen et al., 2011) and TargetP v1.1 (Emanuelsson et al., 2000). Transmembrane helices were analyzed using TMHMM v2.0 (Krogh et al., 2001). Glycosylphosphatidylinositol (GPI) modification sites were predicted by big-PI predictor (Eisenhaber et al., 1999). Extracellular secreted proteins were identified by a combination of characters including the presence of a functional signal peptide and the absence of a trans-membrane domain and GPI modifications (Sperschneider et al., 2015). Potentially secreted proteins with unknown functions (cannot be annotated by Swiss-Prot) were identified as putative effectors. Putative effectors which show no significant homology to known proteins from species outside the genus *Coniella* (BLASTp, *E*-value cut-off of 1e–05) were considered as Genus-specific candidate effectors.

Genes encoding carbohydrate-active enzymes were identified by the hmmscan program in the HMMER 3.0 package against the family-specific HMM profiles of CAZymes downloaded from the dbCAN database (Yin et al., 2012). The resulted file hmmscan was parsed by the hmmscan-parser scripts provided by dbCAN. Gene clusters related to secondary metabolite biosynthesis were identified using the antibiotics and secondary metabolite analysis shell database (antiSMASH v.4.0.2) (Weber et al., 2015).

### Transcriptome Analysis and Quantitative RT-PCR

The agar plug (6 mm diameter) with *C. diplodiella* mycelium was inoculated onto PDA plates covered with or without the ground leaf homogenates from *V. vinifera* “Manicure Finger” or *V. davidii* accession 0940 under aseptic condition, and incubated at 28°C for 6 days (Figure 4). Then, fungal samples were harvested, immediately frozen in liquid nitrogen and stored at −80°C. Total RNA was extracted from six fungal samples with two biological replicates for each of the three different treatments using TRIzol reagent following manufacturer’s instructions (Invitrogen). The quality of RNA was evaluated using Agilent 2100 Bioanalyzer (Agilent, United States). The RNA-seq library construction and sequencing were performed in BGI company using Illumina HiSeq X Ten platform with 150 bp paired-end (PE) mode. Raw RNA-seq data was filtered to remove adaptor sequences and low-quality reads using a BGI internal software SOAPnuke (Chen et al., 2018). The clean reads were mapped to the *C. diplodiella* genome using Hisat2 software (Kim et al., 2015). Transcript assembly was performed using Bowtie 2 (Langmead and Salzberg, 2012) and gene expression levels were calculated based on fragments per kilobase of transcript per million fragments mapped (FPKM) using RSEM (Li and Dewey, 2011). Differential expression analysis was carried out using DEGseq2 (Wang et al., 2010).

For quantitative RT-PCR, the cDNAs were synthesized from 1 μg of total RNA using FastKing RT Kit with DNase (Tiangen Biotech, Beijing, PRC). The gene-specific primers were designed using Primer3Plus software and were listed in Supplementary Table S12. PCR reactions with the Roche FastStart DNA Master SYBR Green I reagent were performed on Roche LightCycler480 instruments with the following procedure: 95°C for 5 min, followed by 45 cycles of 95°C for 10 s, 60°C for 10 s, and 72°C for 20 s. The actin gene of *C. diplodiella* was used as the internal control. The relative gene expression level was analyzed by the 2^–ΔΔCT^ method (Livak and Schmittgen, 2001). Primers used for qRT-PCR were listed in Supplementary Table S12. The data of RNA-seq has been deposited in NCBI Short Read Archive database (SRA accession: PRJNA657740).

### Transient Expression Analysis of Candidate Effectors in *Nicotiana benthamiana*

Candidate effector genes (without putative signal peptide sequence, and an ATG start codon was added to initiate translation) were amplified from cDNA library using PrimeSTAR HS DNA Polymerase (Takara) and were ligated into the PVX vector pGR106. The primers used for vector construction were listed in Supplementary Table S13. Constructs verified by DNA sequencing were introduced into *Agrobactrium tumefaciens* (GV3101) cells containing the helper plasmid pJIC SA_Rep. Agroinfiltration assays were carried out according to the described method (Dou et al., 2008). Briefly, *Agrobacterium* cells carrying pGR106-effector or the respective control plasmids (empty pGR106 and pGR106-Bax) were cultured in LB (Luria-Bertani) medium containing kanamycin (50 μg/ml) overnight at 28°C with agitation. Bacterial cells were harvested by centrifugation (4,000 g, 2 min), washed with 10 mM MgCl_2_ twice and resuspended in MMA buffer (10 mM MgCl_2_, 10 mM MES, 100 μM acetosyringone, pH 5.6). The optical density of cell suspension was adjusted to an OD_600_ of 0.4 and incubated at room temperature for 2–3 h before infiltration. Fully expanded leaves from 5 to 6 weeks old *Nicotiana benthamiana* plants were used for agroinfiltration. Symptoms were observed in the following 3–7 days.

## Results and Discussion

### Genome Assembly and Annotation

The genome of *C. diplodiella* was sequenced by PacBio long-read single molecule real-time (SMRT) technologies to 145-fold coverage with an estimated genome size of 40.93 Mb and GC content of 49.79% (Table 1). The sequencing data (6,321,978,582 bp reads) were *de novo* assembled using CANU (Koren et al., 2017) into 13 contigs with an N50 length of 3.99 Mb, one of which corresponds to the mitochondrial genome with a length of 0.2 Mb. The other 12 contigs constitute the nuclear genome with a total size of 40.73 Mb. We identified 9,403 protein-coding gene models from genome assembly by combining *ab initio* gene prediction and homology-based methods. The average protein-coding gene length is 1,754 bp, with an average of 2.73 exons per gene and an average exon length of 571 bp. Furthermore, RNA-seq analysis of transcripts from *in vitro C. diplodiella* cultures supports the expression of 9,115 genes in the growth conditions tested (Figure 5). Both the estimated genome size and gene number of *C. diplodiella* are comparable to that of its relative *Coniella lustricola*. The completeness of the assembled gene space was evaluated by the benchmarking universal single-copy orthologs (BUSCO) method (Simão et al., 2015) using the Ascomycota dataset. This study identified 1,284 (97.6%) complete and single copy eukaryotic conserved protein-coding sequences (Supplementary Table S1), supporting a high completeness of the assembled *C. diplodiella* genome. Additionally, 311 tRNA and 57 rRNA genes were predicted in the genome (Table 1).

**TABLE 1:** Main features of *Coniella diplodiella* (Speg.) isolate WR01 genome.

***Coniella diplodiella***	
Estimated genome size (Mb)	40.93
Sequencing coverage	145 ×
Number of contigs	13
Contig N50 (Mb)	3.99
Repeat rate (%)	12.74%
GC content (%)	49.79
Number of genes	9,403
Average gene length (bp)	1,754
Average exons per gene	2.73
Average exon length (bp)	571
tRNA	311
rRNA	57

In total, 9,134 (97.14%) predicted genes could be annotated by homology search against multiple databases (Supplementary Tables S2, S3). Of them, 9,125 (97.04%) and 6,557 (69.73%) genes were annotated by NCBI non-redundant protein database (NR) and Swiss-Prot database, respectively. In addition, NR annotation showed 85.99% genes of *C. diplodiella* sharing homology with those of *C. lustricola* (Raudabaugh et al., 2017). Gene Ontology (GO) terms were assigned to 4,219 (44.87%) of the predicted protein-coding genes, including molecular function (3,386 genes), biological process (2,645 genes) and cellular component (1,293 genes) categories (Supplementary Table S3). The most enriched GO terms in the biological process were “single-organism process,” “metabolic process,” and “cellular process” terms (Supplementary Figure S1). KEGG analysis categorized 3,578 (38.05%) genes into 185 pathways (Supplementary Table S3). Among the 34 KEGG subclasses from 5 main classes, the “Global and overview maps,” “Carbohydrate Metabolism,” and “Translation” were the top three subclasses (Supplementary Figure S2). By the KOG (Eukaryotic Orthologous Groups) mapping, 2,870 (30.52%) genes were classified into 26 KOG categories (Supplementary Figure S3).

Repeat sequences were identified using several softwares. The length of total repetitive sequences was 5,182,685 bp, corresponding to 12.74% of the assembled *C. diplodiella* genome (Supplementary Table S4). The percentage of repetitive sequences in *C. diplodiella* is comparable to that of *Valsa mali* genome (14.05%) (Yin et al., 2015). The most abundant repetitive element is the long terminal repeat (LTR) element Gypsy (7.76%), followed by DNA transposons (1.75%), simple repeats (1.58%), non-LTR retrotransposon LINEs (0.49%) (Supplementary Table S5).

### Orthologous Families and Phylogenetic Relationship Analysis

**FIGURE 1:**
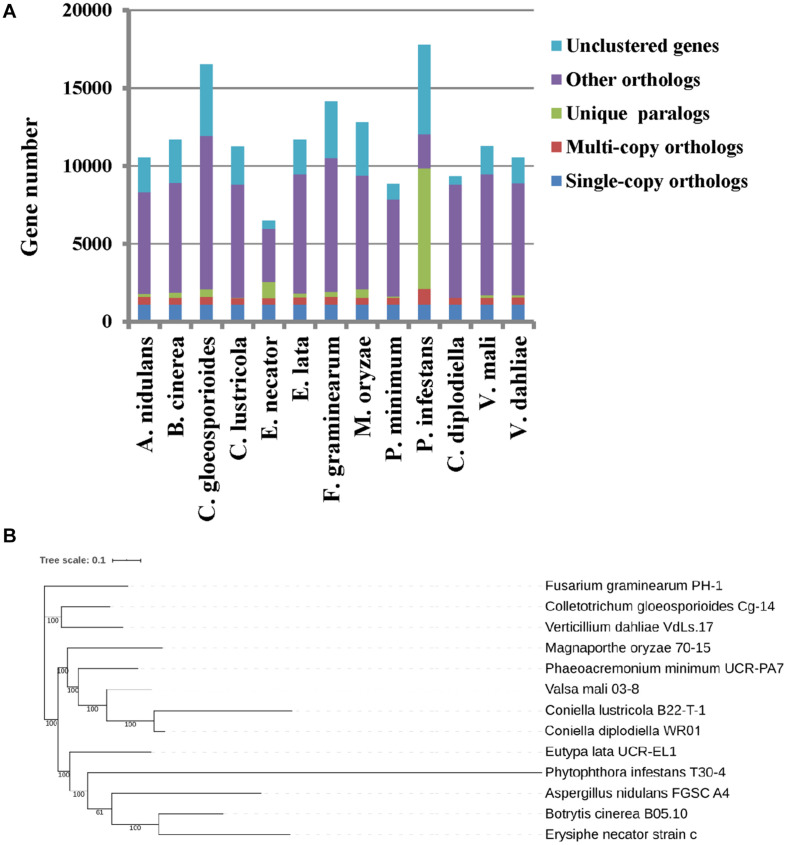
Orthologous gene family and phylogenetic relationship between *C. diplodiella* and 12 other fungi. **(A)** Statistic analysis of the shared and distinct orthologs. **(B)** A maximum likelihood phylogenetic tree was constructed by RaxML based on single-copy orthologous genes.

**FIGURE 2:**
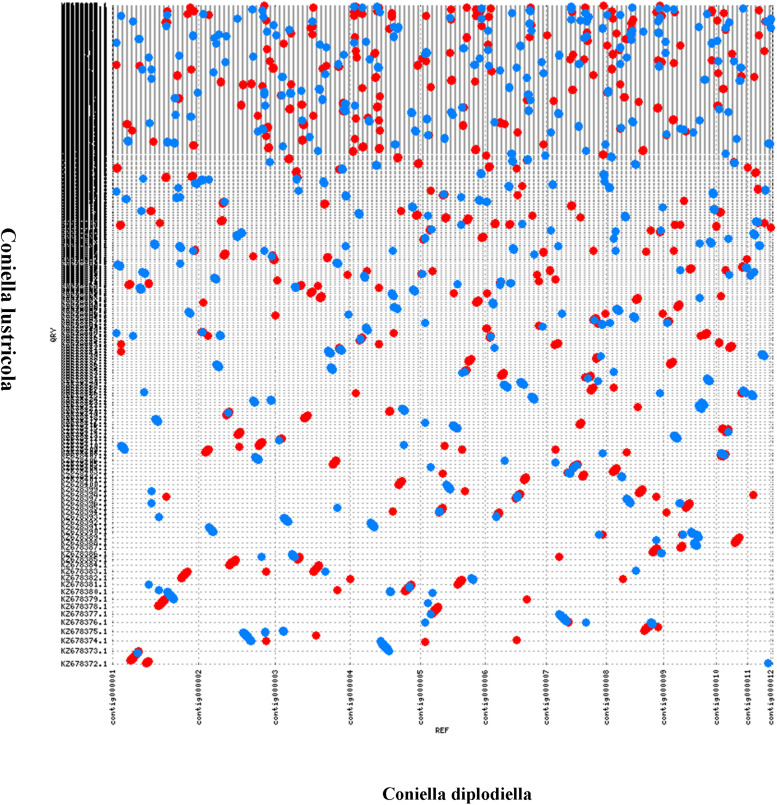
Genome synteny analysis between *C. diplodiella* and *C. lustricola* by MUMmer.

The predicted proteome of *C. diplodiella* was compared to 12 other filamentous fungi with different lifestyles. OrthoMCL analysis showed that *C. diplodiella* shared 8,823 orthologs with the other 12 fungi species, and 1,105 single-copy orthologous genes were conserved among all fungi analyzed (Figure 1A). The phylogenetic tree was constructed by RaxML using single-copy orthologous genes. Phylogenetic analysis revealed that *C. diplodiella* is evolutionally close to *Coniella lustricola* (Figure 1B; Raudabaugh et al., 2017), a non-pathogenic fungus that mainly feeds on plant detritus. Furthermore, we performed synteny comparison between *C. diplodiella* and *C. lustricola*. Genome colinearity comparison revealed a high sequence identity between them (Figure 2).

### Secretome and Putative Effectors

**FIGURE 3:**
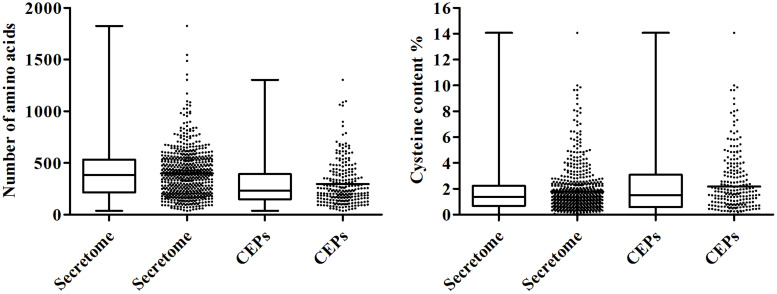
Box-plot shows sequence length and cysteine content of CEPs compared with those of the total secretome.

The pathogenic fungi can deliver distinct sets of secreted proteins into the host-pathogen interface to facilitate its infection during plant-pathogen interactions. Using a combination of softwares including SignalP 4.1, TargetP 1.1, TMHMM 2.0, and big-PI Predictor (Sperschneider et al., 2015; Jones et al., 2018), we predicted 608 genes encoding putative secreted proteins, accounting for 6.47% of *C. diplodiella* proteome (Supplementary Table S6). The proportion of predicted secreted proteins relative to the total proteome is similar to those of other fungal pathogens (5–10%) (Lo Presti et al., 2015). Functional annotation showed that 88% of the secreted proteins have significant homologs outside the genus *Coniella* in GenBank NR database (*E*-value 1e–05) (Supplementary Table S3). Functional enrichment analysis showed that proteins involved in carbohydrate metabolism are significantly overrepresented in the *C. diplodiella* secretome (Supplementary Table S3). Some secreted proteins called effectors can function in the apoplast or translocate into plant cells to manipulate plant immunity (Dou and Zhou, 2012). Because most of the effectors identified so far have low sequence similarity to known proteins (Franceschetti et al., 2017), here candidate effectors proteins (CEPs) were defined as secreted proteins that have no homologs in Swiss-Prot database. Among the 608 secreted proteins of the *C. diplodiella* genome, we identified 246 CEPs, of which 72 proteins are *Coniella*-specific. Comparison analysis of amino acid sequences revealed that CEPs have shorter amino acid sequence length relative to other secreted proteins, with an average of 296 amino acids (Figure 3). In addition, they are cysteine-rich (2.18%) (Figure 3). These are the common properties for known fungal protein effectors (Jones et al., 2018). The identification of the effector repertoire of *C. diplodiella* will provide insights into its virulence function on host plants.

**FIGURE 4:**
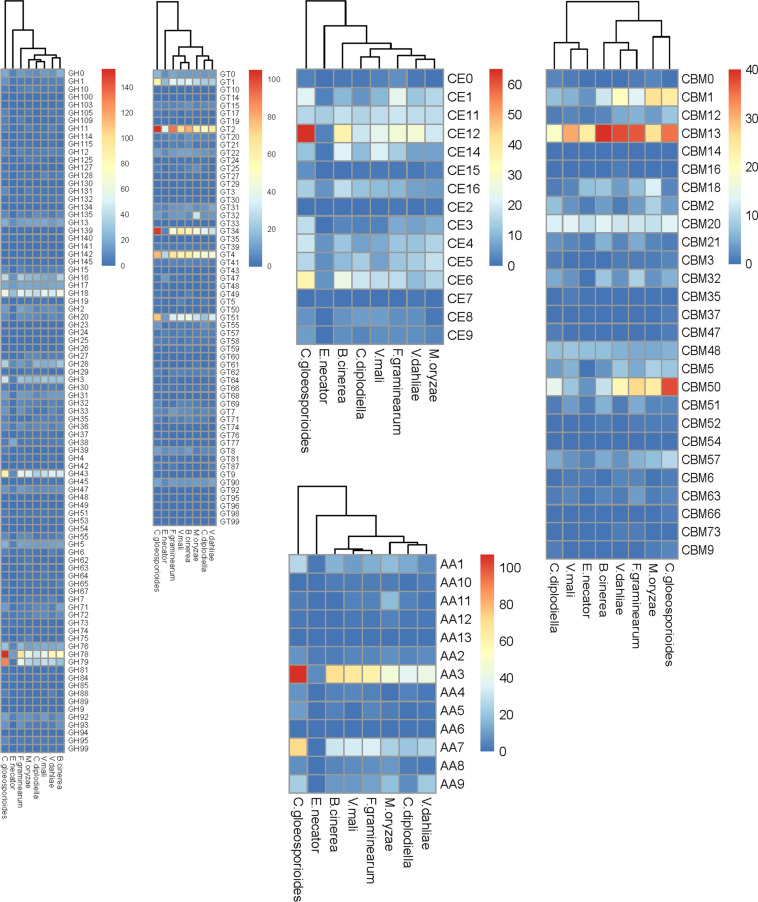
Comparison of carbohydrate-active enzymes between *C. diplodiella* and seven other fungal species. *C. diplodiella, Coniella diplodiella*; *E. necator, Erysiphe necator*; *C. gloeosporioides, Colletotrichum gloeosporioides*; *B. cinerea, Botrytis cinerea*; *V. mali, Valsa mali*; *M. oryzae, Magnaporthe oryzae*; *F. graminearum, Fusarium graminearum*; *V. dahliae, Verticillium dahliae*; GH, glycoside hydrolase; GT, glycosyltransferase; PL, polysaccharide lyase; CE, carbohydrate esterase; AA, auxiliary activity family; CBM, carbohydrate-binding module family.

**FIGURE 5:**
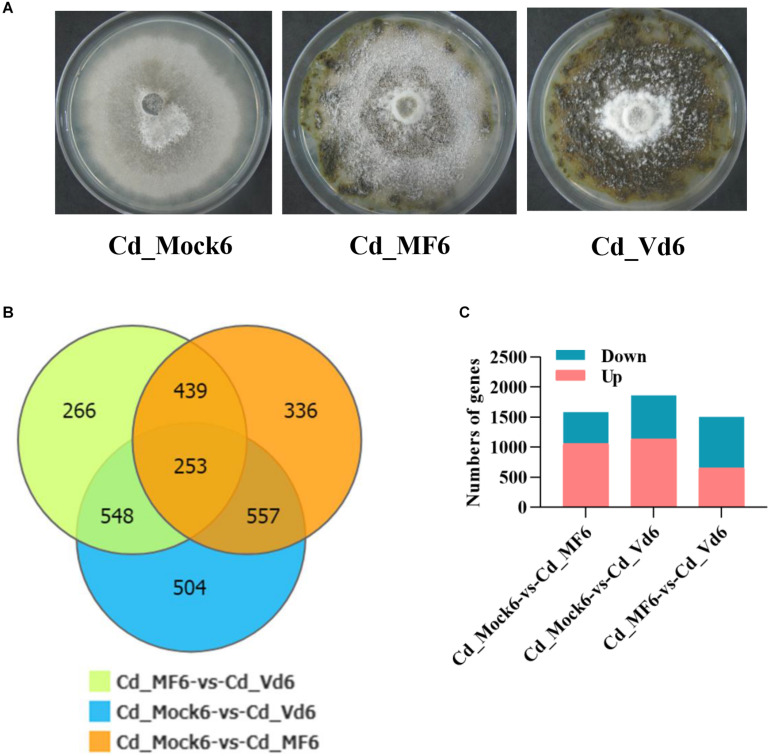
Statistics analysis of differentially expressed genes (DEGs) in *C. diplodiella* in response to susceptible and resistant grapevine host feeding. **(A)** Growth of *C. diplodiella* on potato dextrose agar (PDA) (Cd_Mock6), and PDA overlaid with leaf homogenates from susceptible *V. vinifera* “Manicure Finger” (Cd_MF6) or with that from resistant *V. davidii* accession 0940 (Cd_Vd6) for days at 28°C. **(B)** Venn diagram of DEGs of *C. diplodiella* with different treatments (Mock, MF, or Vd). **(C)** Number of up and down regulated genes of *C. diplodiella* with different treatments (Mock, MF, or Vd).

### Secondary Metabolism Gene Clusters

Fungal secondary metabolites including non-ribosomal peptides, polyketides, NRPS-PKS hybrids, indole alkaloid, and terpenes are widely involved in the responses of fungus to environment stimuli and interaction with other organisms (Brakhage, 2013; Pusztahelyi et al., 2015). Biosynthetic pathways for secondary metabolites contain the backbone enzymes and several decorating enzymes and their corresponding genes tend to be co-regulated at the transcriptional level and clustered in the chromosome (Brakhage, 2013; Massonnet et al., 2018). The genome of *C. diplodiella* contains a total of 39 secondary metabolite gene clusters, including type 1 polyketide synthase (T1PKS, 15 clusters), non-ribosomal peptide synthetases (NRPS, three clusters), type 1 PKS-NRPS hybrids (four clusters), terpene synthases (three clusters), dimethylallyl tryptophan synthases (DMATS, one cluster), one T3 PKS and 13 other clusters (Supplementary Table S7). There are 21 and four genes predicted to encode polyketide synthase and non-ribosomal peptide synthetases, respectively. The numbers of PKS and NRPS genes in *C. diplodiella* are less than those in *V. mali* (Yin et al., 2015). Furthermore, many of these PKS and NRPS genes were surrounded by genes related to cytochrome P450 monooxygenase, dehydrogenase/reductase, alpha/beta-hydrolase and transporters (Supplementary Table S7). However, none of the predicted SM clusters showed high homology to the known clusters in other fungi. To our knowledge, there are no reports regarding bioactive secondary metabolite identified from *Coniella*-species, except for *Coniella fragariae* (Yu et al., 2018). Among the 423 putative secondary metabolism (SM) genes, the transcriptional expression of 414 SM genes was detected by RNA sequencing. Furthermore, comparing with that grown on PDA agar, 106 SM genes were significantly up-regulated in *C. diplodiella* mycelia by leaf tissue homogenates from the susceptible *V. vinifera* “Manicure Finger” grapevine (Supplementary Figure S4C), suggesting that these genes may participate in fungal infection. Of them, several up-regulated genes encode the polyketide synthases, including Cdip_03501, Cdip_04510, Cdip_04513, and Cdip_08045. They are considered as priority candidate genes for future study of secondary-metabolite biosynthesis. The PKS Cdip_08045 is orthologous to the conidial pigment polyketide synthase PfmaE in *Pestalotiopsis fici*, which is involved in melanin biosynthesis (Zhang et al., 2017a). Pigments produced by fungi can involve in fungal development, pathogenesis, and protect them from detrimental environments such as oxidative stress and UV irradiation. In *Botrytis cinerea*, deletions of *PKS12 and PKS13 genes* blocked spore pigmentation production (Schumacher, 2016). Similarly, deletion of PKSs in *Alternaria alternata* caused melanin deficiency and blocked spore formation (Wenderoth et al., 2017). The polyketide synthase gene cluster identification and gene expression profile analysis in this study support the possibility that melanin pigment may contribute to the *C. diplodiella*’s virulence. Moreover, many necrotrophic plant pathogens of the Dothideomycete class can produce phytotoxic metabolites that are required for pathogenicity (Stergiopoulos et al., 2013). The functional characterization of SM genes and the chemical entity of potential secondary metabolites from *C. diplodiella* await further investigation.

### Carbohydrate-Active Enzymes

For successful colonization and infection, phytopathogenic fungi can produce an array of carbohydrate-active enzymes (CAZymes) to break down plant cell wall polysaccharides and derive nutrition from their hosts. The repertoire of CAZymes provides insights into its nutritional mode and infection mechanisms (Zhao et al., 2013). The *C. diplodiella* genome encoded 1,141 putative CAZymes. These CAZymes contain 507 glycoside hydrolases (GHs), 313 glycosyltransferases (GTs), 115 carbohydrate esterases (CEs), 98 auxiliary activities (AAs), 14 polysaccharide lyases (PLs) and 94 carbohydrate-binding modules (CBMs) and were categorized into 160 distinct families (Supplementary Table S8). Similarly, a total of 1,179 CAZymes including 531 GHs, 322 GTs, 125 CEs, 96 AAs, 13 PLs, and 92 CBMs were predicted in *C. lustricola* genome (Supplementary Table S8). Beside *C. lustricola*, the CAZyme profiles of *C. diplodiella* are close to that of other necrotrophic plant pathogens, such as *Botrytis cinerea*, *Valsa mali*, and *Verticillium dahliae* (Supplementary Table S8).

Plant cell walls are composed primarily of pectins, celluloses, hemicelluloses, lignins, and proteins. The GHs are the largest family involved in the carbohydrate degradation process. The GH class contributes the most catalytic enzymes to the degradation of lignocelluloses. The most genes encoding for the lignocellulose degrading enzymes belong to the GH3, GH16, GH18, GH28, GH43, GH78, GH79 family in *C. diplodiella* genome (Figure 4 and Supplementary Table S9). Fungal pathogens usually encode a large number of pectin-degrading enzymes including pectinlyase, pectatelyase, pectinesterase, and polygalacturonase to efficiently digest pectin. Polygalacturonases (family GH28) and pectinesterases (family CE8) catalyze the de-esterification of pectin to pectate and methanol. Most fungi contain only a small number (no more than 8) of pectinesterases, which may play a critical role in pectin degradation during pathogen infection. The genome of *C. diplodiella* contains 25 polygalacturonase genes and nine pectinesterases genes, suggesting a potential ability for pectin degradation (Figure 4 and Supplementary Table S9). In addition, there are abundant GT classes related with hemicellulose (GT34) and chitin (GT2) degradation and pectin-degrading enzymes (PL1 and PL3) in *C. diplodiella* genome (Figure 4 and Supplementary Table S9).

The *C. diplodiella* contains no radical-copper oxidases of the family AA5, an essential enzyme for lignin degradation, while several AA5 genes exist in other necrotrophic and hemibiotrophic pathogens examined, except for *V. mali* (Figure 4 and Supplementary Table S9). There are fewer lytic polysaccharide monooxygenases (LPMOs) of the family AA9, which cleave cellulose chains in synergic with classical cellulases, and also smaller number of family CBM1 members in *C. diplodiella* genome, compared with the cereal pathogens *C. graminicola* and *M. oryzae* (Figure 4 and Supplementary Table S9). Plant cuticle composed of a cutin polymer matrix is an effective physical barrier against the majority of pathogens. Therefore, plant fungal pathogen needs to produce cutinases in the early infection stages to launch infection (Dickman et al., 1989; Lu et al., 2018). Considering that the initial infection of *C. diplodiella* often requires a pre-existing wound site, it is surprising that the high number of cutinase genes of family CE5 exists in *C. diplodiella* genome (Figure 4 and Supplementary Table S9). RNA-seq analysis showed that most the cutinase-encoding genes are expressed in very low level or not detected (Supplementary Table S11), suggesting that they don’t involve in initial infection, and their expression may require a special stimulus or only in special infection stage.

### RNA-Seq Analysis of Transcriptome Changes of *C. diplodiella* in Response to Host Grapevine Feed

Because the successful infection of *C. diplodiella* usually require wounds on the grapevine, and its destructive necrotrophic lifestyle (Chethana et al., 2017), this makes it difficult to recover enough fungus samples for examining gene expression *in planta*. Alternatively, we incubated the fungus on PDA supplemented with resistant or susceptible grapevine leaf homogenates to partially mimic the physiological response of *C. diplodiella* to its host plant. The growth of *C. diplodiella* was inhibited by resistant *Vitis davidii*, compared with that grown on PDA or susceptible *V. vinifera* (Figure 5A).

**FIGURE 6:**
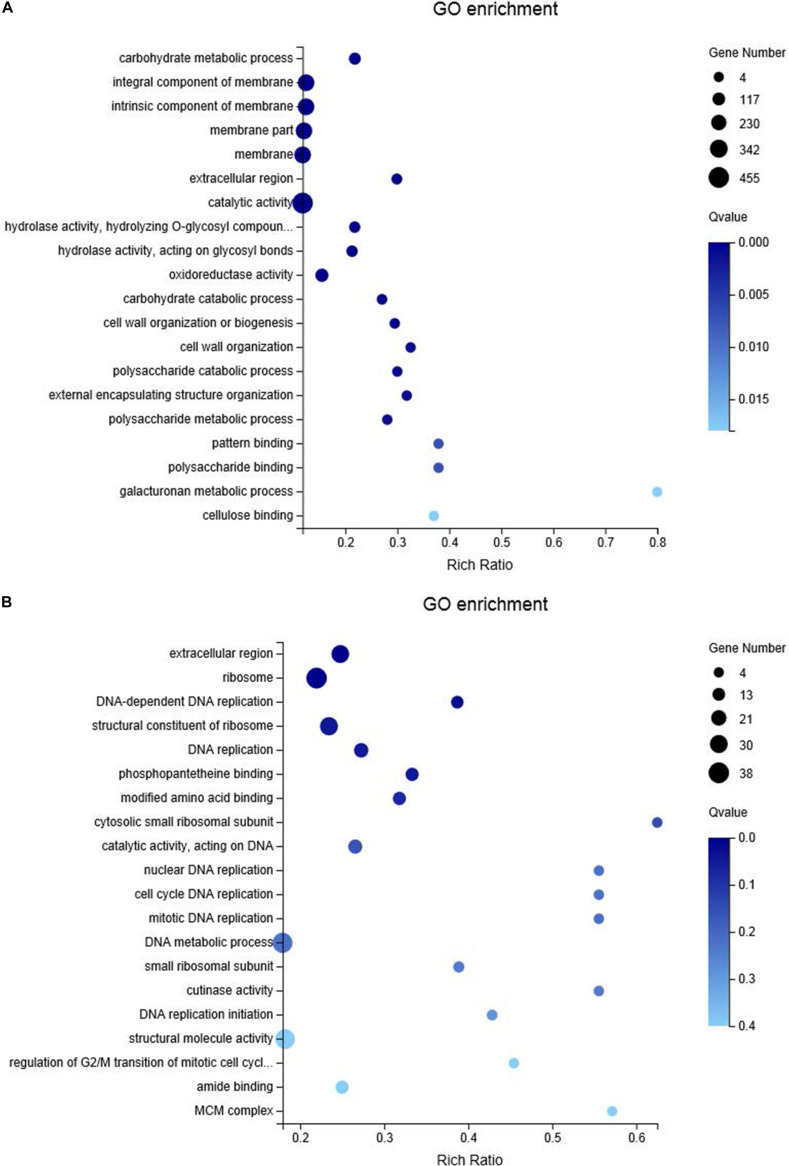
Bubble diagram of top 20 enriched GO terms of up-regulated genes in *C. diplodiella* in response to susceptible **(A)** and resistant **(B)** grapevine host feeding. *X* axis represents the Rich Ratio, which meaning the ratio of selected gene number annotated to a particular item to the total number of genes in this item in one species. The calculating formula is Rich Ratio = Term Candidate Gene Num/Term Gene Num. *Y* axis represents GO term. The size of the bubbles indicates the number of genes annotated to a GO term. And the color represents *Q*-value of enrichment. The deeper the color, the smaller the *Q*-value.

For RNA-sequencing, about 6.6 Gb cleaned data and 44 Mb clean reads were generated for each cDNA library (Cd_Mock6-1, Cd_Mock6-2, Cd_MF6-1, Cd_MF6-2, Cd_Vd6-1, Cd_Vd6-2) (Supplementary Table S9). The clean reads Q30 value was about 91% and approximately 85.53–88.9% of the clean reads were mapped to the *C. diplodiella* genome (Supplementary Table S9). The expression levels of all the transcripts were estimated by FPKM (fragments per kilo-base of exon per million fragments mapped) using RSEM (Li and Dewey, 2011). Overall, a total of 2,861 DEGs and 253 common DEGs were identified among three different treatments (Figure 5B). There were 1,585 DEGs (528 up-regulated and 1,057 down-regulated) in Cd_Mock6-vs-Cd_MF6, 1,861 DEGs (727 up-regulated and 1,135 down-regulated) in Cd_Mock6-vs-Cd_Vd6, and 1,506 DEGs (854 up-regulated and 652 down-regulated) in Cd_MF6-vs-Cd_Vd6 (Figure 5C). Quantitative RT-PCR was performed with nine selected genes to validate the accuracy of the gene expression profiles derived from RNA-seq data. The relative expression levels of all the nine genes were overall consistent with those obtained from RNA-seq data (Supplementary Figure S5). Gene Ontology enrichment analysis of the up-regulated genes in Cd_Mock6-vs-Cd_MF6 showed that predominant DEGs are enriched in catalytic activity, membrane-related process and carbohydrate metabolic process (Figure 6A), while the riched GO terms of the up-regulated genes in Cd_Mock6-vs-Cd_Vd6 belong to the ribosome, DNA replication and extracellular region (Figure 6B). These results suggest that resistant *V. davidii* may possess special genes/metabolites for resisting *C. diplodiella* infection.

### Functional Analysis of Putative Effectors

**FIGURE 7:**
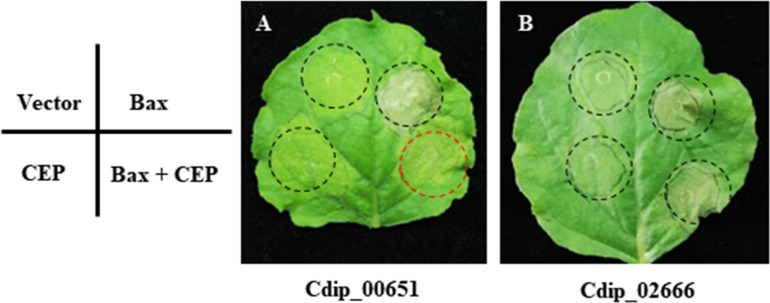
Effects of transient expression of candidate effector proteins on Bax-induced programmed cell death (PCD) in *N. benthamiana* using agroinfiltration. *N. benthamiana* leaves were infiltrated with *Agrobacterium tumefaciens* cells carrying pGR106 empty vector or the Bax gene, or candidate effector genes. For cell death suppression assays, co-expression of CEPs and Bax by mixing an equal volume of cells (OD600 = 0.8). Both Cdip_00651 **(A)** suppressed Bax-induced PCD, while other CEPs tested, such as Cdip_02666 **(B)**, failed to block Bax-induced PCD. One representative result was showed in figure, and two independent experiments with six biological replicates for each experiment were performed with similar results.

Effector proteins secreted by plant pathogens are key virulence factors that can suppress plant defense responses and modulate host cell physiology to promote infection (Lo Presti et al., 2015). When transiently expressed in plant cells, the pro-apoptotic mouse protein BAX (BCL2-associated X) can trigger programmed cell death (PCD) that closely resemble hypersensitive response in plants (Lacomme and Santa Cruz, 1999). Therefore, the ability to suppress BAX-triggered PCD has been used as a powerful tool for initial screening of candidate effectors (Wang et al., 2011; Li et al., 2015). To gain information on the function of putative effectors in plant-pathogen interaction, we randomly selected 43 candidate effector genes (the detailed information was listed in Supplementary Table S6) for transient expression in *N. benthamiana*. When co-infiltrated with BAX, Cdip00651 (here we named as *Coniella diplodiella* effector 1, CdE1) shows significant suppression of BAX-triggered PCD, but all other 42 candidate effectors do not (Figure 7), suggesting that CdE1 involves in plant immune suppression. CdE1 encodes a 91 amino acid protein with a predicted secretory signal peptide (amino acids 1–31) and contains no recognizable functional domain. The possible role of CdE1 in virulence and/or avirulence function needs to be explored by constructing *CdE1* gene deletion and overexpression *C. diplodiella* mutants. In general, biotrophic and hemibiotrophic pathogens deliver effectors to interfere with PCD. In contrast, necrotrophic pathogens secrete effectors to promote plant cell death (Wang et al., 2014). For instance, the ToxA protein of *P. tritici-repentis* triggers cell death in wheat by targeting the host chloroplastic protein ToxABP1 (Manning et al., 2007). In this study, we identified one effector that suppresses BAX-triggered PCD among the 43 candidate effectors tested, but no one was found to induce cell death on non-host *N. benthamiana*. Given that necrotrophic effectors are usually host-specific, the necrosis-inducing activity of these CEPs needs to be further determined on hosts. As more effectors from necrotrophic pathogens were identified, the effectors with cell death-suppressing activity have been found. Several effectors of *V. mali* were found to suppresses Bax-induced PCD, in which both VmEP1 and VmPxE1 were demonstrated to contribute to the virulence of *V. mali* (Li et al., 2015; Zhang et al., 2018). Phytotoxin oxalic acid (OA) is a key virulence factor in the necrotrophic fungus Sclerotinia sclerotiorum and host interaction (Kabbage et al., 2013). OA-deficient mutants display non-pathogenic phenotype and trigger autophagic cell death, indicating that autophagy act in host defense against this pathogen and OA contributes to the suppression of autophagy (Kabbage et al., 2013). Therefore, we can assume that necrotrophic pathogens also employ effectors to suppress defense responses manifested as an PCD. Functional characterization of the host targets of CdE1 in grapevine will help to ascertain the roles of CdE1 in *C. diplodiella*-grapevine interaction.

## Conclusion

Although grape white rot is one of the main fungal diseases in viticulture regions in China and widely distributed among most grape cultivation areas of the world, there is limited knowledge of its pathogenic mechanisms. The genome sequence and annotation of *C. diplodiella* revealed the genomic characteristics. Genome comparative analysis provided evidence for evolutionary relationships between *C. diplodiella* and *C. lustricola*. *C. diplodiella* contains a large number of genes encoding carbohydrate-active enzymes, consistent with other necrotrophic pathogens and its relative *C. lustricola*. As an essential feature for the necrotrophic pathogens is the use of special secondary metabolites as virulence factors, we identified numerous secondary biosynthetic gene clusters. None of them share similarity with known biosynthetic gene clusters, indicating that *C. diplodiella* may produce novel secondary metabolites. During plant-pathogen interaction, fungal effectors are crucial regulator of both pathogen virulence and plant immunity, therefore, identification of the target of effectors in plant host will provide insights into the way how pathogen interfere with host immunity for the success of infection. Further functional analysis of the pathogenesis-related candidate genes will improve our understanding of the interaction between *C. diplodiella* and grapevine.

## Data Availability Statement

The data of RNA-seq has been deposited in NCBI Short Read Archive database (SRA accession: PRJNA657740).

## Author Contributions

YZ, CL, and RL conceived and designed the experiments. RL, YZ, and LS analyzed the data. YW, JJ, and XF helped to prepare biological materials and data analysis. RL and PL did the RT-PCR, gene cloning, and gene transient expression. RL wrote the draft manuscript. YZ, YW, and CL revised the manuscript. All authors have read and approved the final manuscript for submission.

## Conflict of Interest

The authors declare that the research was conducted in the absence of any commercial or financial relationships that could be construed as a potential conflict of interest.

## Supplementary Material

Supplementary Figure 1 GO classification of total predicted genes in *C. diplodiella*.

Supplementary Figure 2 KEGG pathway classification of total predicted genes in *C. diplodiella*.

Supplementary Figure 3 KOG function classification of total predicted genes in *C. diplodiella*.

Supplementary Figure 4 The expression pattern of total DEGs **(A)** and genes for carbohydrate enzymes **(B)**, and secondary metabolite synthesis **(C)** in response to susceptible and resistant grapevine host feeding.

Supplementary Figure 5 RT-PCR validation of expression levels of 9 DEGs identified by RNA-seq. Values in left vertical axis represents relative expression levels of selected DEGs quantified by qRT-PCR in three different treatments. The right vertical axis shows the fragments per kilo-base per million mapped reads (FPKM) values from RNA-seq. For qRT-PCR data, the means and SD of two biological replicates were shown.

Supplementary Table 1 BUSCO analysis of *C. diplodiella* contigs.

Supplementary Table 2 Statistics of *C. diplodiella* gene annotation.

Supplementary Table 3 Summary of *C. diplodiella* gene annotation.

Supplementary Table 4 Repeatitive sequence statistic of *C. diplodiella*.

Supplementary Table 5 Summary of repeat element types in the *C. diplodiella* genome.

Supplementary Table 6 Characteristics of secretome in *C. diplodiella*.

Supplementary Table 7 Inventory of secondary metabolism gene clusters in *C. diplodiella.*

Supplementary Table 8 Statistics of CAZymes family in different fungi.

Supplementary Table 9 The gene numbers of CAZymes subfamily in different fungi.

Supplementary Table 10 The overall quality and mapping of RNA-seq reads from *C. diplodiella* with three different treatments.

Supplementary Table 11 Expression of putative cutinase-coding genes in *C. diplodiella*.

Supplementary Table 12 Primers used for qRT-PCR.

Supplementary Table 13 Primers used for gene cloning of selected candidate effector genes.
