# Supplementary figures and images for: Genome Assembly and Transcriptome Analysis of the Fungus Coniella diplodiella During Infection on Grapevine (Vitis vinifera L.)
Source: Front Microbiol. 2021 Jan 11;11:599150. doi: 10.3389/fmicb.2020.599150 (PMC7829486; doi:10.3389/fmicb.2020.599150)

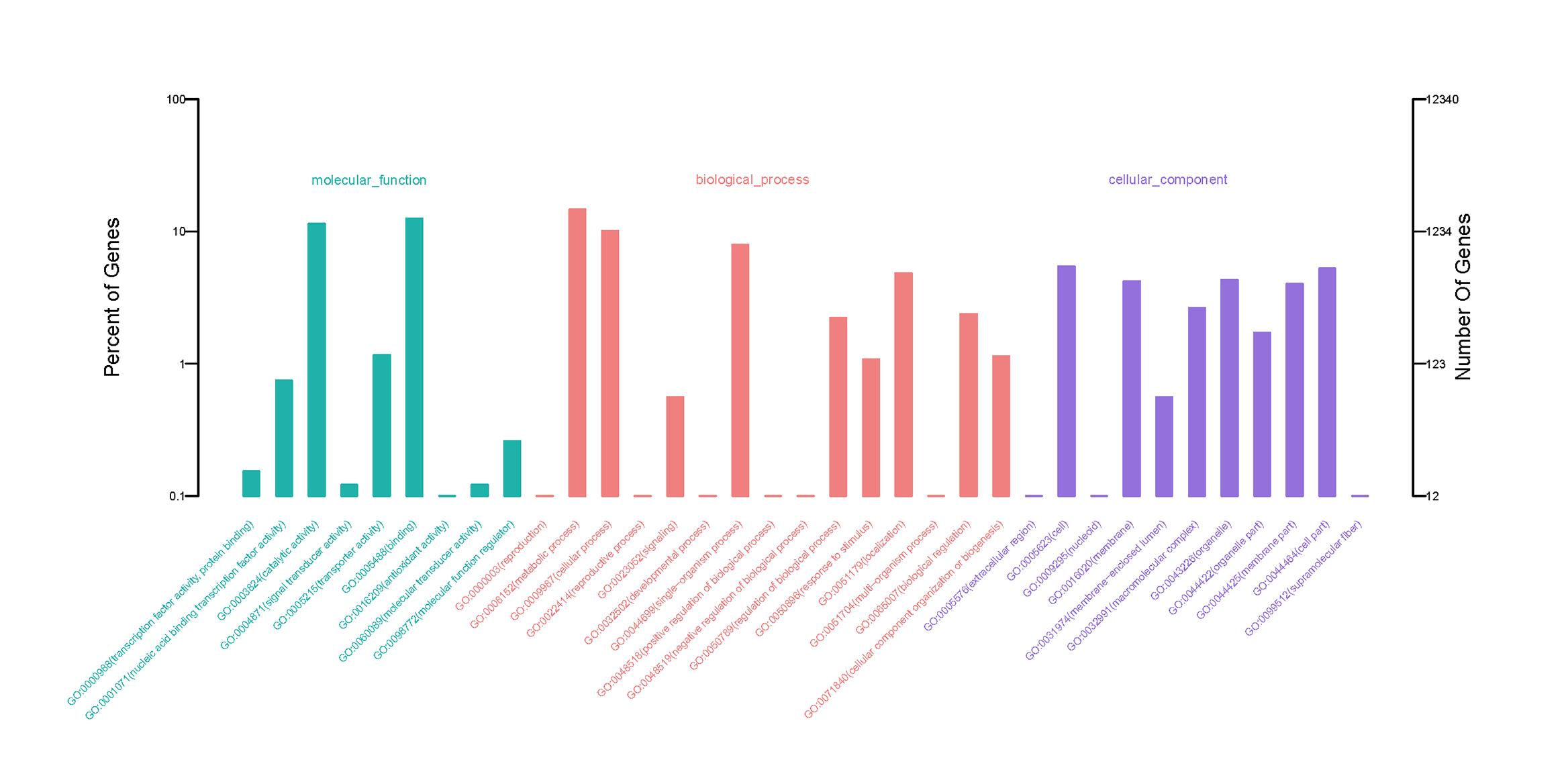

Supplement: Supplementary Figure 1 — GO classification of total predicted genes in C. diplodiella. [file Image_1.TIF]

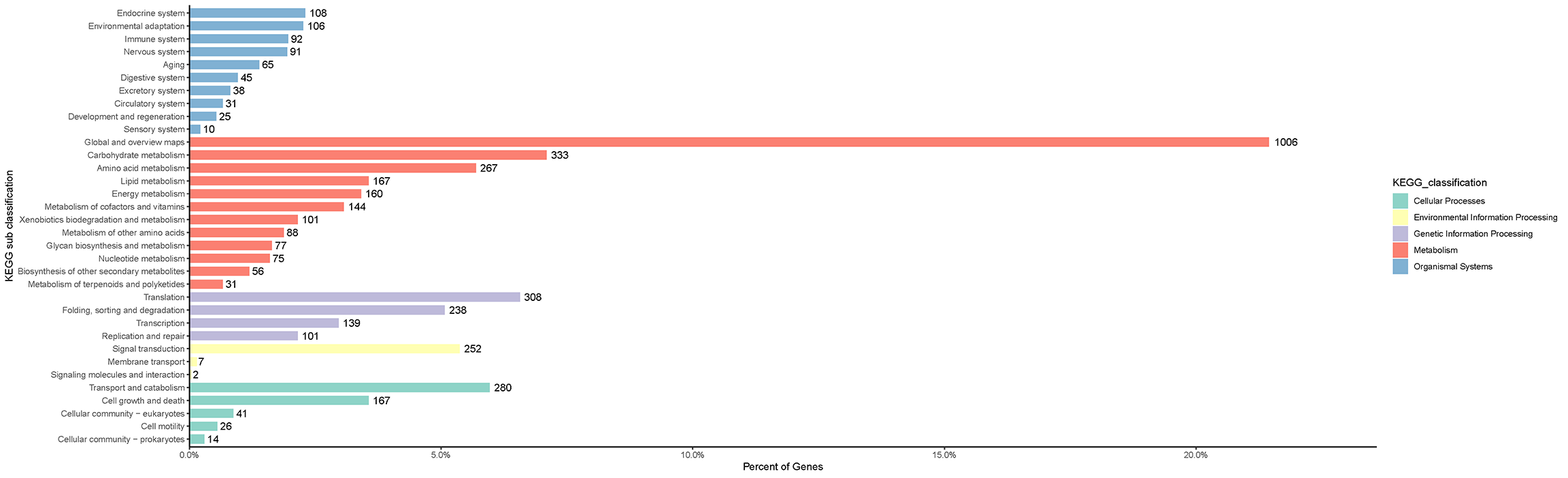

Supplement: Supplementary Figure 2 — KEGG pathway classification of total predicted genes in C. diplodiella. [file Image_2.TIF]

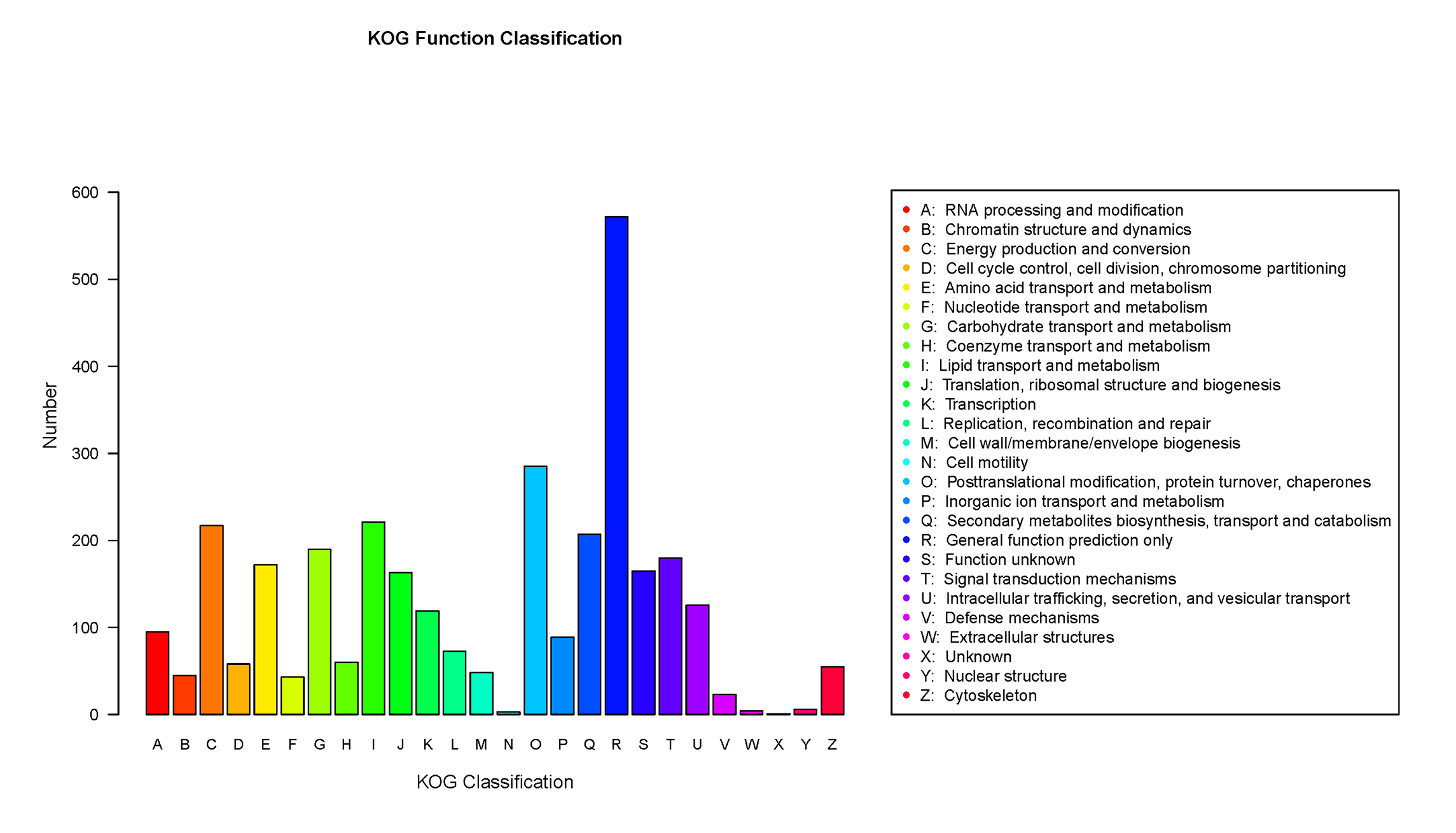

Supplement: Supplementary Figure 3 — KOG function classification of total predicted genes in C. diplodiella. [file Image_3.TIF]

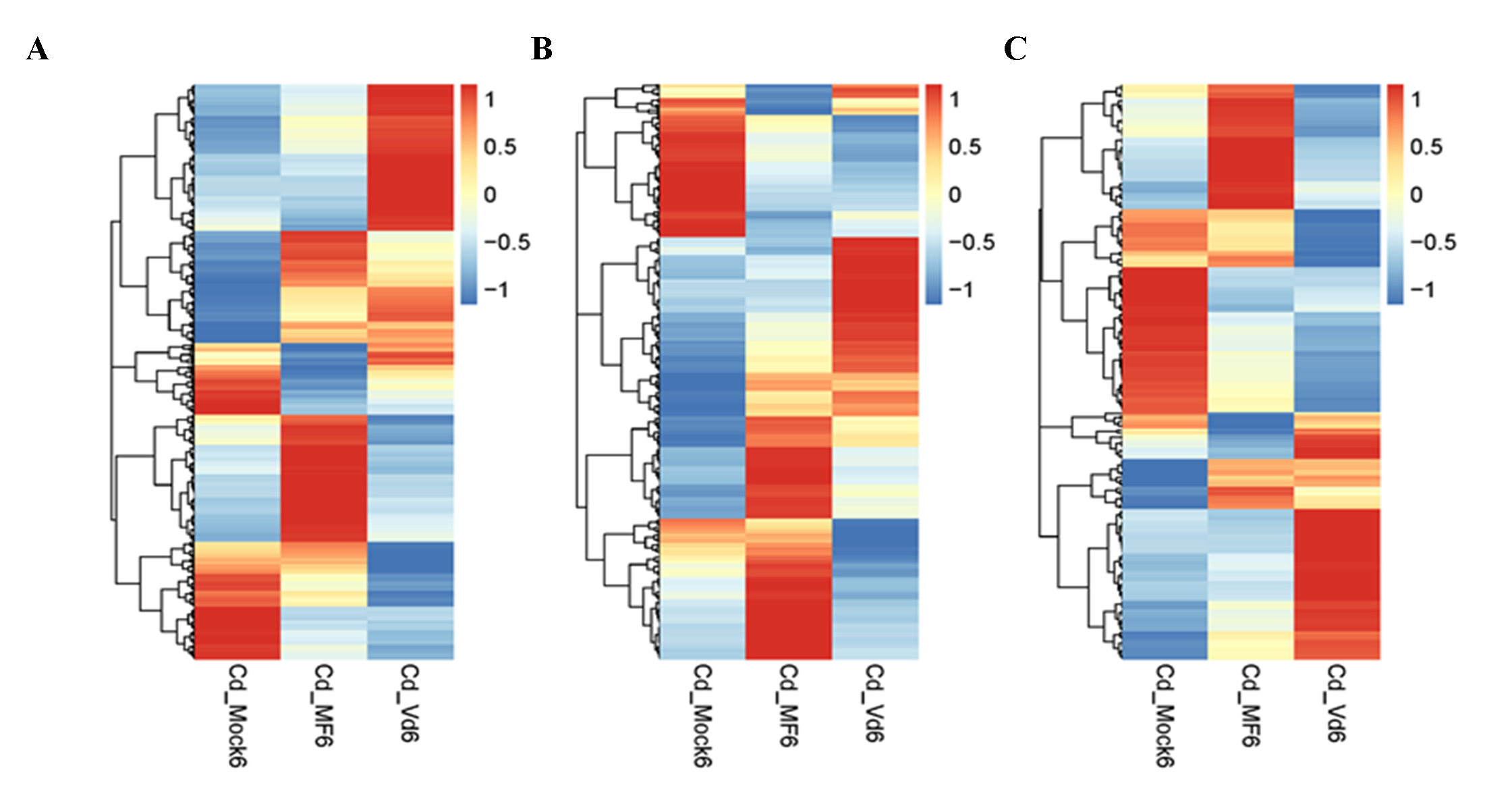

Supplement: Supplementary Figure 4 — The expression pattern of total DEGs (A) and genes for carbohydrate enzymes (B), and secondary metabolite synthesis (C) in response to susceptible and resistant grapevine host feeding. [file Image_4.TIF]

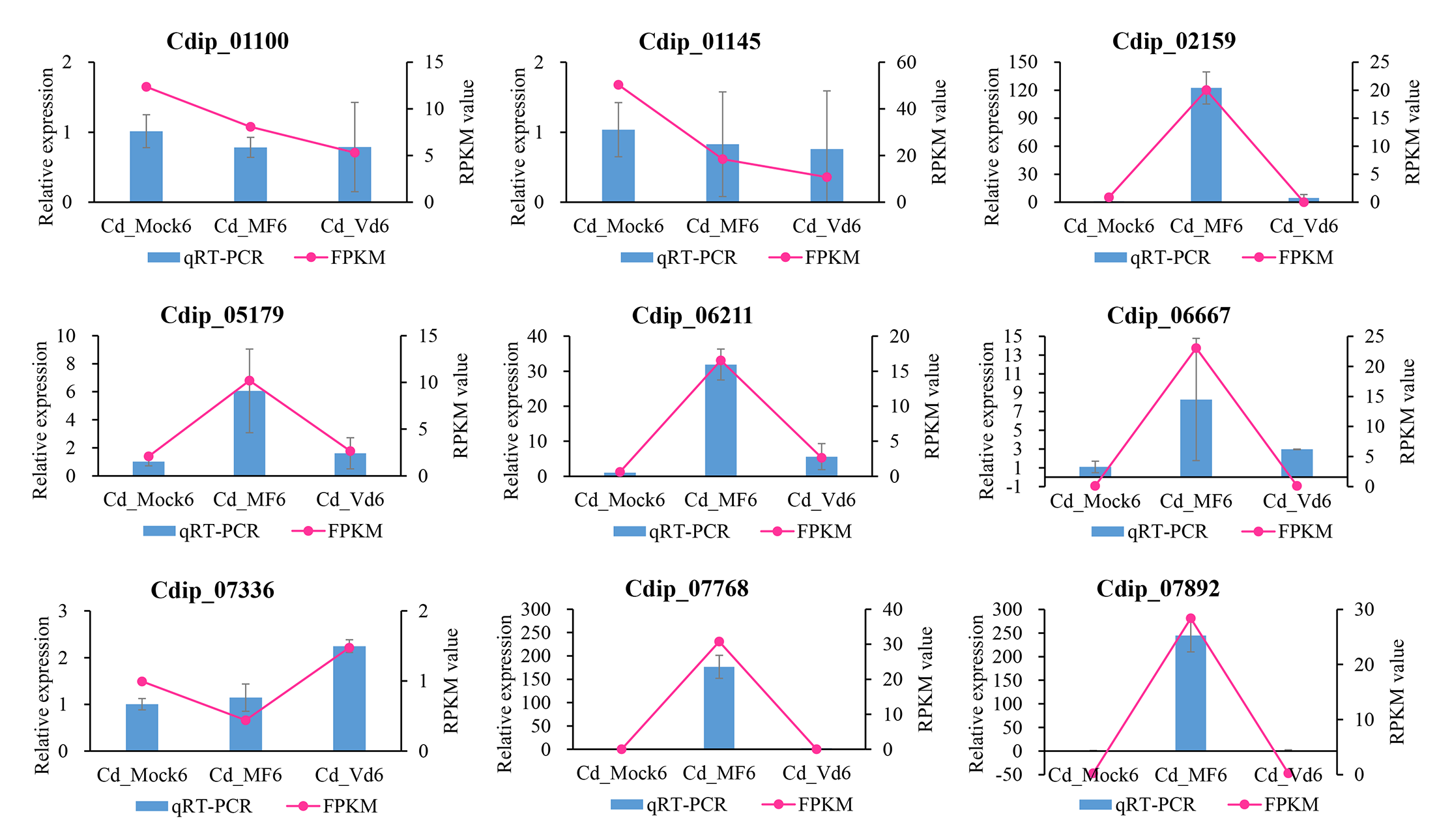

Supplement: Supplementary Figure 5 — RT-PCR validation of expression levels of 9 DEGs identified by RNA-seq. Values in left vertical axis represents relative expression levels of selected DEGs quantified by qRT-PCR in three different treatments. The right vertical axis shows the fragments per kilo-base per million mapped reads (FPKM) values from RNA-seq. For qRT-PCR data, the means and SD of two biological replicates were shown. [file Image_5.TIF]
